# ApoB100/LDLR-/- Hypercholesterolaemic Mice as a Model for Mild Cognitive Impairment and Neuronal Damage

**DOI:** 10.1371/journal.pone.0022712

**Published:** 2011-07-26

**Authors:** Carlos Ramírez, Saleta Sierra, Inmaculada Tercero, Jose Antonio Vázquez, Antonia Pineda, Tatiana Manrique, Javier S. Burgos

**Affiliations:** BioPharma Division, Neuron BPh, Granada, Spain; Boston University School of Medicine, United States of America

## Abstract

Recent clinical findings support the notion that the progressive deterioration of cholesterol homeostasis is a central player in Alzheimer's disease (AD). Epidemiological studies suggest that high midlife plasma total cholesterol levels are associated with an increased risk of AD. This paper reports the plasma cholesterol concentrations, cognitive performance, locomotor activity and neuropathological signs in a murine model (transgenic mice expressing apoB100 but knockout for the LDL receptor [LDLR]) of human familial hypercholesterolaemia (FH). From birth, these animals have markedly elevated LDL-cholesterol and apolipoprotein B100 (apoB100) levels. These transgenic mice were confirmed to have higher plasma cholesterol concentrations than wild-type mice, an effect potentiated by aging. Further, 3-month-old transgenic mice showed cholesterol (total and fractions) concentrations considerably higher than those of 18-month-old wild-type mice. The hypercholesterolaemia of the transgenic mice was associated with a clear locomotor deficit (as determined by rotarod, grip strength and open field testing) and impairment of the episodic-like memory (determined by the integrated memory test). This decline in locomotor activity and cognitive status was associated with neuritic dystrophy and/or the disorganization of the neuronal microtubule network, plus an increase in astrogliosis and lipid peroxidation in the brain regions associated with AD, such as the motor and lateral entorhinal cortex, the amygdaloid basal nucleus, and the hippocampus. Aortic atherosclerotic lesions were positively correlated with age, although potentiated by the transgenic genotype, while cerebral β-amyloidosis was positively correlated with genetic background rather than with age. These findings confirm hypercholesterolaemia as a key biomarker for monitoring mild cognitive impairment, and shows these transgenic mice can be used as a model for cognitive and psycho-motor decline.

## Introduction

In recent years, converging lines of clinical and pathological evidence have indicated a relationship to exist between the deterioration of brain cholesterol homeostasis and the pathophysiology of sporadic AD. Epidemiological studies have shown that hypercholesterolaemia is an early (rather than a late) risk factor for AD, and that elevated plasma concentrations of LDL cholesterol correlate well with the appearance of AD [1], [2], [3], [4]. Patients with familial hypercholesterolaemia (FH) show a particularly high incidence of mild cognitive impairment (MCI), a prodromal stage of abnormal cognitive performance which precedes AD [2], [4]. Patients with FH provide a unique window into the role of cholesterol metabolism in cognition. FH is caused by inherited genetic abnormalities that directly or indirectly affect the function of the LDL receptors (LDLRs) [5]. The LDLR family has also been implicated in the breakdown of synaptic function in AD [6].

A number of authors have analysed the effect of hypercholesterolaemia in animals in terms of Aβ modulation, reporting it linked to the appearance of Aβ deposits in the brains of rabbits [7], [8] and the acceleration of cerebral Aβ deposition in APP-transgenic mice [9], [10]. Although it is clear that dietary and pharmacological alterations of plasma lipid metabolism can influence cognitive impairment and Aβ deposition in AD transgenic animals, it is not yet known whether increases in baseline lipid profiles in non-AD mouse models provoke behavioural deficits or neurodegeneration. Some authors have reported the absence of LDLRs in 6 month-old mice to lead to impaired spatial memory [11], although no aging studies nor cholesterol determinations were undertaken. Other authors report transgenic LDLR-deficient Tg2576 mice to develop high-level hypercholesterolaemia and age-dependent cerebral β-amyloidosis [12]. However, both Tg2576 and LDLR-deficient Tg2576 mice showed impaired learning after Aβ deposition, although the latter showed greater spatial learning deficits. These results leave it unclear whether APP overexpression and/or hypercholesterolaemia truly contribute to cognitive impairment. Moreover, some authors report that plasma cholesterol levels are not significantly altered in classic murine AD models [9] such as Tg-CRND8, APP/PS1 or Tg-SwDI/B, suggesting that these transgenic mice do not replicate the hypercholesterolaemia observed in humans. If hypercholesterolaemia really is a risk factor for cognitive impairment, the optimal animal model in which to study this would be one in which hypercholesterolaemia appears early in life.

The apoB100/LDLR-/- mouse developed by Powell-Braxton et al. [13] mimics FH very well since the hypercholesterolaemia these animals suffer is caused almost entirely by high plasma concentrations of cholesterol-rich apoB100-containing LDL, i.e., the same as that which typically occurs in humans. To test whether hypercholesterolaemia facilitates the progress of MCI to AD, the plasma cholesterol levels, cognitive and locomotor status, and the pathological signs of such progress were monitored in naïve apoB100/LDLR-/- mice at 3 and 18 months of age. The results suggest that hypercholesterolaemia may be an important factor influencing the neuropathological changes and cognitive and psychomotor impairment associated with aging.

## Materials and Methods

### A) Animals

#### Ethics statement

All experiments were undertaken in accordance with the guidelines of the European Union, took place under the supervision of veterinary staff, and were approved by the ethical committees of NEURON BPh (“Comité Ético de Bienestar Animal” or CEBA) and the University of Granada (“Comité Ético de Experimentación Animal” or CEEA). The approval number of the CEEA was Ref. CEEA 133-2007. Additionally, although the Spanish Law does not require it, NEURON BPh evaluated the experimental use of animals and the particular procedures through its ethical committee (CEBA).

#### Experimental procedures

The study animals were 3- and 18-month-old, male, homozygous apoB100/LDLR-/- transgenic mice and male, wild-type C57Bl/6 mice (Charles River, Barcelona, Spain). Male mice were selected because they show higher levels of total and LDL-cholesterol than females [13]. All were housed in an air-conditioned room under a 12L/12D photoperiod until sacrifice, and had free access to water and food. At the moment of sacrifice the mice were deeply anaesthetized with fluorane (2% with O_2_ at 0.2 L/min and air at 1.5 L/min) and blood collected in EDTA-coated tubes following cardiac puncture. The plasma obtained was conserved at −80°C for biochemical determinations. Immediately after blood extraction the animals were perfused intracardially with PBS followed by 3.7% formaldehyde in phosphate buffer. Their brains and aortas were then removed and post-fixed in 3.7% formaldehyde in phosphate buffer.

### B) Plasma cholesterol determinations

Plasma total cholesterol (TC), LDL-cholesterol, HDL-cholesterol, triglycerides (TG) and apoB were measured spectrophotometrically using kits from Biosystems (Barcelona, Spain). Free (FC) and esterified (EC) cholesterol were determined fluorometrically. Briefly, plasma samples were diluted 1/500 in a sodium phosphate monobasic buffer with 0.5% triton X-100 (pH 7). Cholesterol in the concentration range 0.31 to 20 µg/mL was transferred to 96-well plates to produce a fluorometric standard curve; 25 µL of the diluted plasma were also transferred to 96-well plates for the determination of FC. 75 µL of 2-(N-morpholino) ethanesulphonic acid (MES) 0.05 M (pH 6.5) containing cholesterol oxidase (0.5 U/mL), type VI horseradish peroxidase (4 U/mL) and ampliflu red (20 µg/mL) were added to the plates and reactions allowed to proceed for 15 min at 37°C. The fluorescence intensities were measured using a multi-well plate reader equipped with a filter set for excitation and emission at 530 and 580 nm respectively (Infinite 200, Tecan) [14]. Cholesterol esterase (0.8 U/mL) was added to the reaction mixture to measure total cholesterol. Esterified cholesterol was calculated as the difference between TC and FC.

### C) Behaviour testing

#### Evaluation of muscular strength

The grip strength test determines the muscular strength and neuromuscular integration relating to the grasping reflex in the forepaws [15]. Grip strength was assessed using a grip strength meter (Panlab, Barcelona). The strength measurements of young transgenic mice (n = 6), young wild type mice (n = 6), aged transgenic mice (n = 8) and aged wild type mice (n = 8), were measured five times (in succession) by an investigator blinded to genotype. The maximum grip strength values were used for subsequent analysis; data were expressed as force in grams normalized to body weight in kg (g/kg).

#### Motor coordination

Motor coordination was tested using an accelerating rotarod LE8200 apparatus (Panlab, Barcelona). All animals (n = 7 for both aged groups of mice, and n = 6 for both young groups) were given 1 min training before testing and were then placed on the rod and tested from 4 to 40 rpm for a maximum of 120 s. Each animal underwent a minimum of three trials with 10 min intervals between them, during which time the mice were allowed to recover in their home cages. The total time that each mouse was able to stay on the rod was recorded automatically by a trip switch under the floor of each rotating drum, activated by the animal's fall. The average time to fall (fall latency) for three trials was then determined [16].

#### Open field testing

The global locomotor activity of the mice was determined by open field testing as previously described [17]. Tests were performed in an environment chamber that provided white noise and low, indirect lighting. The animals were placed in the centre of the 45×45×40 cm arena (Panlab, Barcelona) and allowed to explore freely for 10 min. The exploration track of each mouse (n = 7 for the aged groups and n = 6 in young groups) was video-recorded. SMART software (Panlab, Barcelona) was used to monitor spontaneous locomotor activity. Periods of immobility were recorded after the first 2 s of a lack activity. Vertical activity was calculated by counting the number of rearings.

#### Memory testing

The long-term memory for different objects, their spatial location and their order of presentation was assessed in a familiar open field (35×45×45 cm) made of red PVC. A video camera was mounted 100 cm above the box to record the trials on a hard disk for later analysis. Diffuse white light provided an illumination density of approximately 3.0 lux at the centre of the open field. Four identical examples of two objects differing in height, colour, shape and surface texture were then presented to the mice. After each test period, the apparatus and objects were thoroughly cleaned with a 70% ethanol solution to remove odour cues.

One week prior to testing, 6 mice per age/genotype group were allowed an exploration period during which they became familiar with the test apparatus (five sessions of 10 min each) and handling procedure. Memory status was analysed using the integrated memory test, following a procedure adapted from a previously described experiment [18] that has been validated in a KA-induced neurodegeneration mouse model [19]. Briefly, the mice underwent two sample trials and a test trial. In the first sample trial each mouse was separately placed in the open field with four identical objects arranged in a triangular configuration and were allowed to explore them for 10 min. After a delay of 50 min each mouse received a second sample trial in which four novel objects were presented; these were arranged in a square configuration. The objects presented in sample trials 1 and 2 were randomly determined for each mouse. After an additional delay of 50 min each mouse underwent a test trial identical to the second, except that two of the objects from sample trial 1 (“old familiar” objects) and two of the objects from sample trial 2 (“recent familiar” objects) were presented. For each mouse, the time spent exploring the object was recorded with a stopwatch. The exploration of an object was assumed to be underway when the mouse approached it and had physical contact with it, either with its vibrissae, snout, or forepaws. Immobility in the vicinity of an object was considered not to be exploratory behaviour. The total time spent exploring the four objects during the sample and test trials was scored. The ratio between the time spent exploring “old familiar” objects and “recent familiar” ones shows the temporal memory to be functional if the value is higher than 1 (differences in exploration time were considered significant when p<0.05). The ratio between the time spent exploring displaced and unmoved “old familiar” shows the spatial memory to be functional if the value is higher than 1 (differences in exploration time were considered significant when p<0.05). All experiments were performed during the light phase of the day.

### D) Immunohistochemistry

Paraffin-embedded sections (5 µm) of aorta and brain were deparaffinated and dehydrated for immunohistochemical labelling. The latter was performed by incubating the samples overnight with each appropriately diluted primary antibody in 10% blocking solution at 4°C and then with the appropriate biotinylated secondary antibody (1∶500) (Jackson ImmunoResearch, Tokyo, Japan) at room temperature for 1 h. The samples were then processed using the Elite ABC Perox kit (Vector Laboratories, Burlingame, CA), and the peroxidase reaction product detected using 3,3′-diaminobenzidinetetrahydrochloride (DAB) (Vector Laboratories). Finally, sections were mounted in DPX (Panreac, Barcelona). Digital bright-field images were captured using a Zeiss Axiovert Z1 (Carl Zeiss, Barcelona, Spain) optical microscope equipped with a colour camera. A mouse MAP2 monoclonal antibody (1∶200) (Sigma-Aldrich, MO) was used to detect microtubule signals [20] and a mouse HNE monoclonal antibody (1∶100) (Jaica, Fukuroi, Japan) to detect oxidative damage by lipid peroxidation [21]. Rabbit anti-glial fibrillary acidic protein (GFAP) polyclonal antibody (1∶5000) (Dako, Glostrup, Denmark) was used to identify the activation and proliferation of astrocytes [22].

### E) Detection of cerebral β-amyloidosis

The ACCUSTAIN® Congo red staining kit (Sigma) was used to stain amyloid deposits following the manufacturer's instructions. Briefly, samples were deparaffinated and hydrated with deionised water. The slides were then stained with Mayer's haematoxylin solution for 10 min, rinsed in tap water for 5 min, and placed in an alkaline sodium chloride solution for 20 min. Samples were then stained with alkaline Congo red solution for 20 min, rinsed in absolute ethanol, cleared in xylene, and mounted. The slides were examined microscopically using regular light to detect amyloid deposits, and their presence confirmed using polarized light to check for apple-green birefringence (a characteristic of these deposits).

For β-amyloid immunodetection, deparaffinated and dehydrated brain sections were incubated overnight with monoclonal anti-β-amyloid (4G8) antibody (1∶500) (Sigma-Aldrich, MO) in blocking solution at 4°C and then with the appropriate biotinylated secondary antibody (1∶500) (Jackson ImmunoResearch, Tokyo, Japan) in PBS at room temperature for 1 h. The samples were then processed using the Elite ABC Perox kit (Vector Laboratories, Burlingame, CA), and the peroxidase reaction product detected using DAB (Vector Laboratories).

### F) Quantification of aortic atherosclerosis

The aortic arch was discarded and the thoracic aorta embedded in paraffin. Sections were then cut at intervals of 5 µm. Slides from representative areas of the artery were stained with Van Gieson stain to assess the presence and severity of atheroma plaque and the characteristics of the media layer. Two independent investigators blind to the study protocol evaluated each section for different characteristics relating to the presence or absence of plaque and the deterioration of the elastic fibres in the media layer. A score of 0–3 (0 perfect condition, 3 extremely deteriorated) was awarded to each slide for 1) the elasticity or aging of fibres, 2) the rupture of fibres, and 3) the loss of the classical structure of the elastic fibres.

### G) Statistical analysis

The mean and SEM were calculated for all ratios and parametric values. One-way analysis of variance (ANOVA) followed by a Newman-Keuls *post-hoc* test was used to compare grip strength and rotarod data between the different age/genotype groups. The two-tailed t test was used to compare the ratios recorded in the integrated memory test against the preset paradigm value of 1. The one-tailed t test was used to compare the ratios recorded for the different genotype/age groups. Significance was set at p<0.05. All statistical analyses were performed using STATISTICA for Windows (Tulsa, OK).

## Results

### A) Plasma determinations

**Figure 1:**
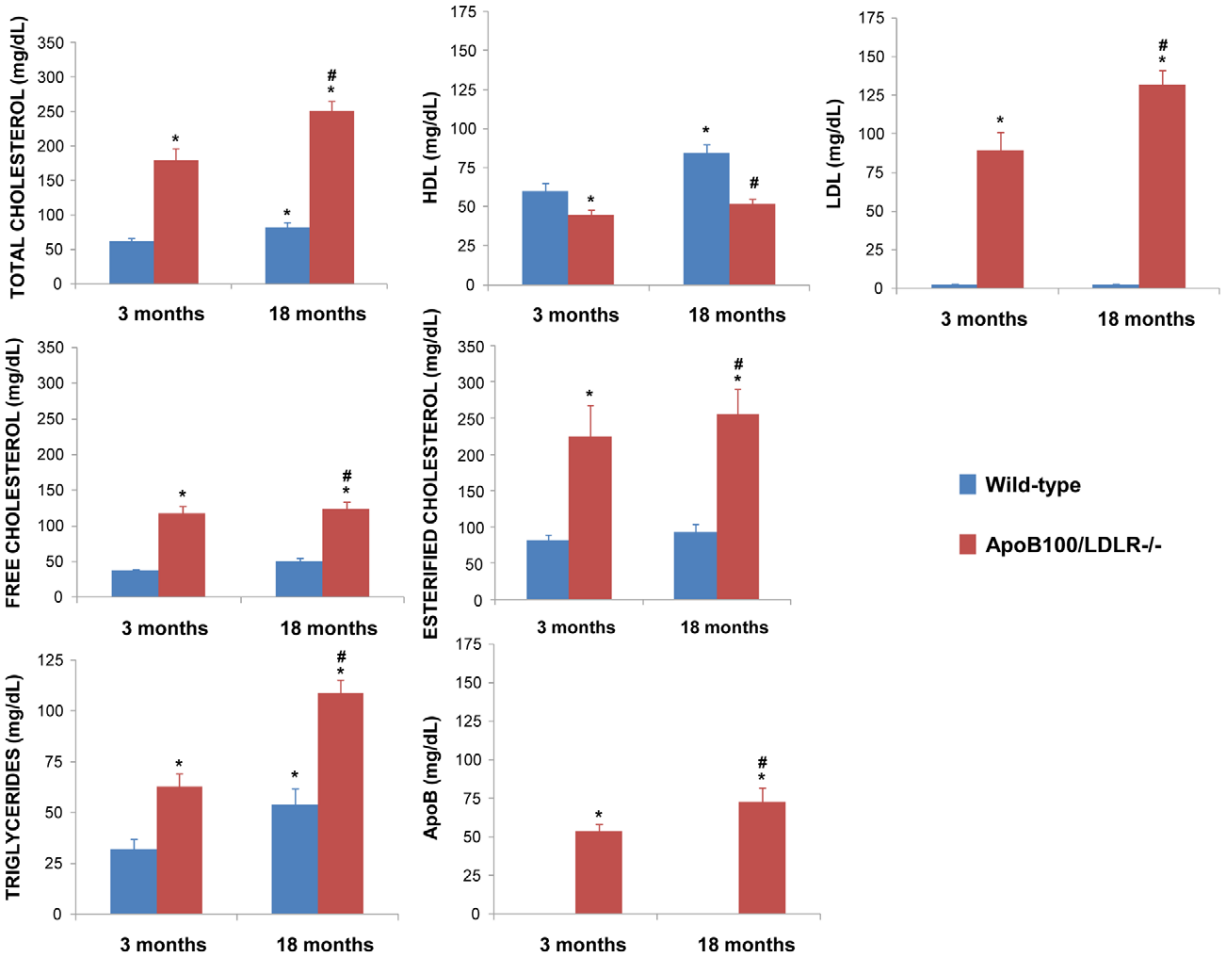
Values of plasma variables in young and aged wild-type and transgenic mice. Total cholesterol, HDL-cholesterol, LDL-cholesterol, apoB and triglycerides were assessed by spectrophotometric methods (blue bar  =  wild-type, red bar  =  transgenic). Free and esterified cholesterol were determined by fluorometric assays. The results, expressed in mg/dL, are means±SEM (n≥6 per group). *Significantly different (P<0.05) compared to 3-month-old wild-type mice; ^#^Significantly different (P<0.05) between wild type and transgenic mice.

Figure 1 shows the results for the lipid variables in the 3 and 18-month-old wild-type and transgenic mice. Plasma TC, FC, EC, LDL-cholesterol, apoB and TG concentrations were significantly higher at both 3 and 18 months of age in the transgenic mice, particularly so for LDL-cholesterol. The transgenic mice also showed lower plasma concentrations of the atheroprotective HDL-cholesterol fraction at both ages. These mice therefore suffer hypercholesterolaemia. Indeed, the young transgenic mice had higher concentrations of TC, FC, EC, LDL and apoB (but not HDL) than the aged wild-type mice, indicating that their genetic background is linked to a more dramatic hypercholesterolaemia than that seen in normal aging.

### B) Analysis of overall locomotor activity

**Figure 2:**
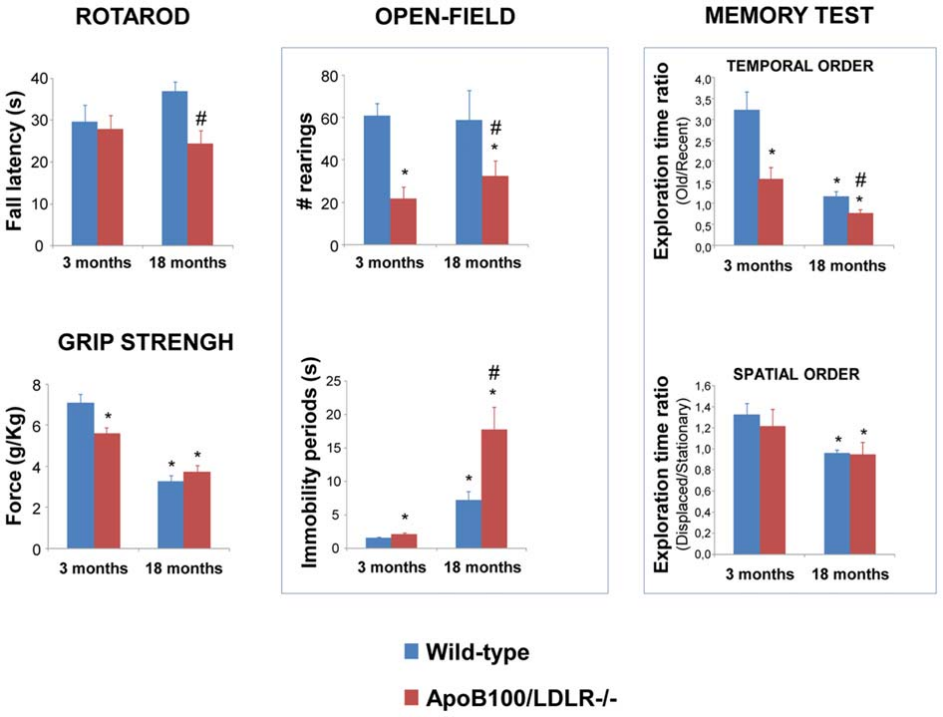
Behavioural phenotyping. Effects of genotype (blue bar  =  wild-type, red bar  =  transgenic) and age on fall latency (in seconds to fall from the accelerating rotarod); grip strength (expressed as g/kg of weight); vertical activity (number of rearings) and period of immobility (in seconds, open field test); and temporal memory (as a ratio) and spatial memory (as a ratio). Data are expressed as means±SEM. *Significantly different (P<0.05) compared to 3-month-old wild-type mice; ^#^Significantly different (P<0.05) between wild type and transgenic mice.

In the rotarod test, the aged transgenic mice showed significantly shorter fall latencies than the aged wild-type mice (p<0.05) (Fig. 2). In the grip strength test, the 3-month-old transgenic animals showed significantly reduced grip strength compared to the young wild-type mice (p<0.05). Aging was involved in the loss of grip strength since both the 18-month-old wild-type and transgenic mice showed significant reductions in the value of this variable compared to 3-month-old wild-type mice (p<0.05). In the analysis of global activity in the open field test, the transgenic mice showed a severe reduction in vertical activity as well as an increase in immobility. The latter increased with age (p<0.05).

### C) Analysis of memory status

After the motor activity tests, cognitive status was assessed in the same animals using the integrated memory test, a paradigm previously optimised to evaluate episodic-like memory in mice [19]. This method includes the analysis of episodic-like memory (temporal and spatial order memory) (Fig. 2). The young wild-type mice showed their temporal memory to be intact, returning ratios significantly greater than the paradigm value, whereas the aged wild-type mice showed a significant reduction in both types of memory (p<0.05). With respect to temporal memory, both the 3- and 18-month-old transgenic mice returned significantly lower ratios than wild-type mice of the same ages (p<0.05). In additional analysis, the 18-month-old transgenic mice, showed significantly poorer spatial memory values than their 3-month-old counterparts (p<0.05). In summary, young transgenic mice showed a temporal memory decline similar to that seen in aged wild-type mice, indicating that the formers' hypercholesterolaemia is likely involved in memory impairment. Spatial memory decline, in contrast, appears to be more related to ageing.

### D) Determination of aortic atherosclerosis

**Figure 3:**
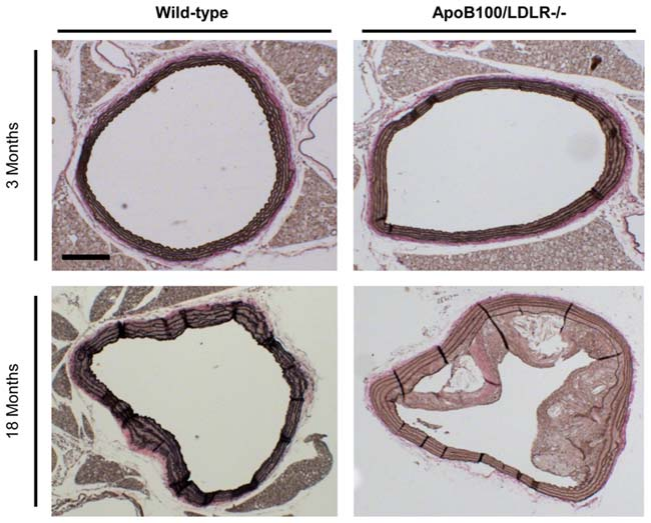
Structure of the thoracic aorta of young and aged wild-type and transgenic mice. Six sections (stained with Van Gieson stain) from different areas of the aorta were evaluated by two blinded observers, scoring for the deterioration of the elastic fibres in the media layer. The absence or presence of atheroma plaque was also independently examined. Scale bar: 100 µm.

To test whether the transgenic mice were more susceptible than wild-type mice to atherosclerosis, signs of aortic atherosclerosis were sought in both young and aged animals. The aged wild-type mice and the young transgenic mice showed a deterioration of the middle layer (Fig. 3). The 18-month-old transgenic mice, however, had evident atheroma plaques of considerable size, with vast necrotic cores and with thin fibrous caps about to break. The high LDL-cholesterol concentrations of these mice, acting synergistically with ageing, would therefore appear to be involved in the development of such atherosclerosis and eventually non-stable plaques. The severe atherosclerotic process underway might be associated with poor brain irrigation, leading to impaired cognitive function. Interestingly, neither the young transgenic nor wild-type animals showed any significant aortic atherosclerotic lesions. Aortas showing deterioration of the elastic fibres (mainly the loss of their classic structure and rupture) were much more commonly seen in 18-month-old transgenic mice (overall mean score 1.5±0.3). The young transgenic mouse aortas showed a marked alteration of the classical structure (overall mean score 0.6±0.2), while in the 18-month-old wild-type mice the fibres showed a loss of elasticity and their clear aging (total score, 0.9±0.3). The 3-month-old wild-type mice showed intact arteries (overall mean score 0.0±0.0).

### E) Neuropathology

#### Neuritic dystrophy

**Figure 4:**
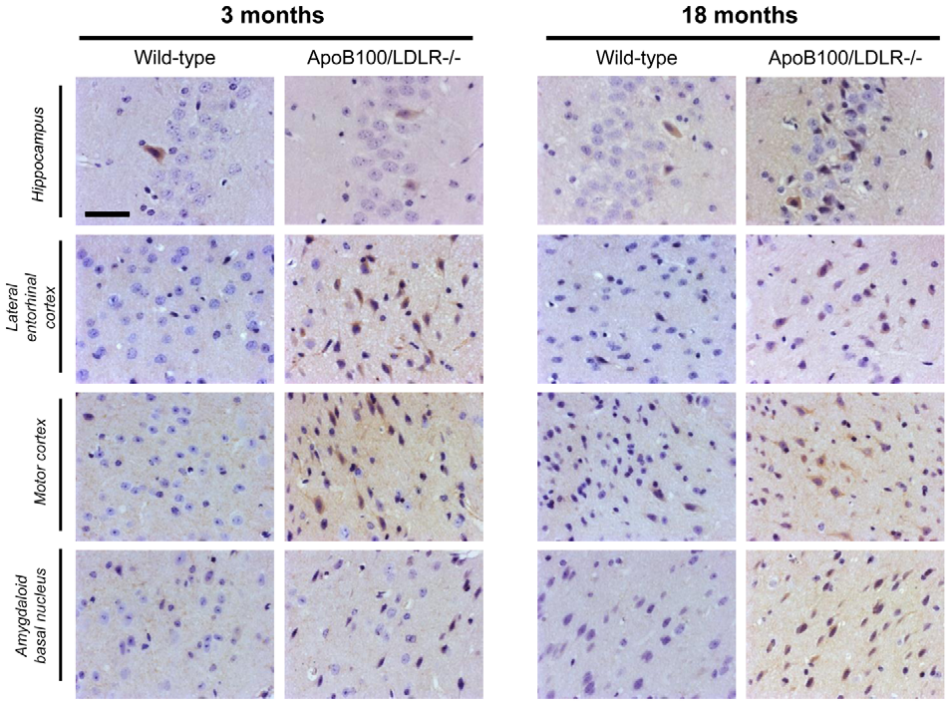
MAP2 staining in young and aged wild-type and transgenic mice. MAP2 staining of coronal sections (1.78 mm from the bregma) of the left (contralateral) hemisphere of a mouse brain from each of the age/genotype groups. Hippocampus and three areas of the cortex showing several nuclei of the temporal lobe cortex, such as the lateral entorhinal, ectorhinal, perirhinal and temporal-association cortices, the primary and secondary somatosensory cortices, lateral parietal association cortex and visual cortex, and the cortical amygdaloidal nucleus and amygdalohippocampal area. Scale bar: 25 µm.

MAP2 monoclonal antibodies were used to determine the degree of neuritic dystrophy as a marker of neuronal damage (Fig. 4). Compared to the wild type mice of the same age, the dendrites and axons in the lateral entorhinal cortex and amygdaloidal basal nucleus of the 3-month-old and 18-month-old transgenic mice showed a clear reduction in the MAP2 signal. However, no difference was seen between the transgenic mice of either age. Thus, staining is genotype and not age-dependent. No loss of the MAP2 stain was observed in the 3-month-old or 18-month-old wild-type mice, whose microtubule networks showed dense and uniform labelling. However, the dentate gyrus of the hippocampus of the 18-month-old wild-type mice showed a strong reduction in dendrite – but not axon - MAP2 staining compared to the young wild-type mice. This pattern of reduction in dendrite MAP2 staining was also observed in both the young and aged transgenic mice. These results suggest that the transgenic genotype causes the deterioration of the hippocampus, a very important area involved in memory.

#### Oxidative damage

**Figure 5:**
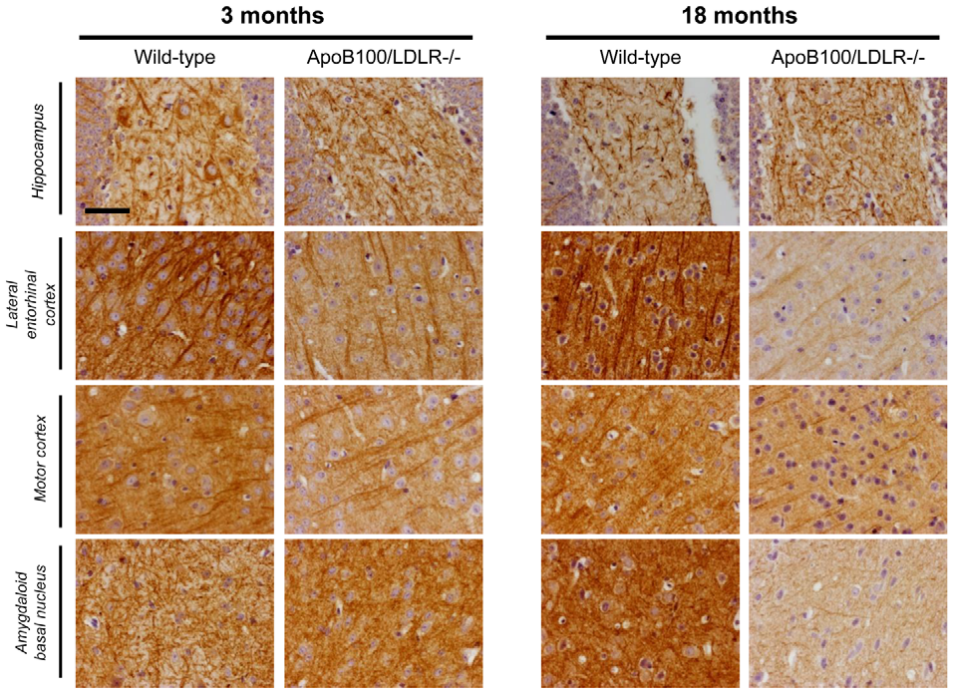
Lipid peroxidation in young and aged wild-type and transgenic mice. HNE stains of coronal sections (1.68 mm from bregma) of the left (contralateral) hemisphere of a mouse brain from each of the age/genotype different groups. The hippocampus and three areas of the cortex are represented, showing several nuclei of the temporal lobe cortex, such as the lateral entorhinal, the ectorhinal, perirhinal and temporal-association cortices, the primary and secondary somatosensory cortices, the lateral parietal association cortex and visual cortex, and the cortical amygdaloidal nucleus and amygdalohippocampal area. Scale bar: 25 µm.

Staining of the HNE-conjugated proteins were used to study the degree of lipid peroxidation (a marker of oxidative damage) in the brain. A large number of deeply stained neurons were seen in various brain regions in the transgenic mice. Staining was stronger in the hippocampus and amygdala of the 18-month-old transgenic mice than in the 3-month-old transgenic mice (Fig. 5). Very few isolated neurons were stained in the brains of wild-type mice. These results show the strong relationship between the transgenic genotype and oxidative damage in the brain; no such clear relationship is seen with ageing.

#### Astrogliosis

**Figure 6:**
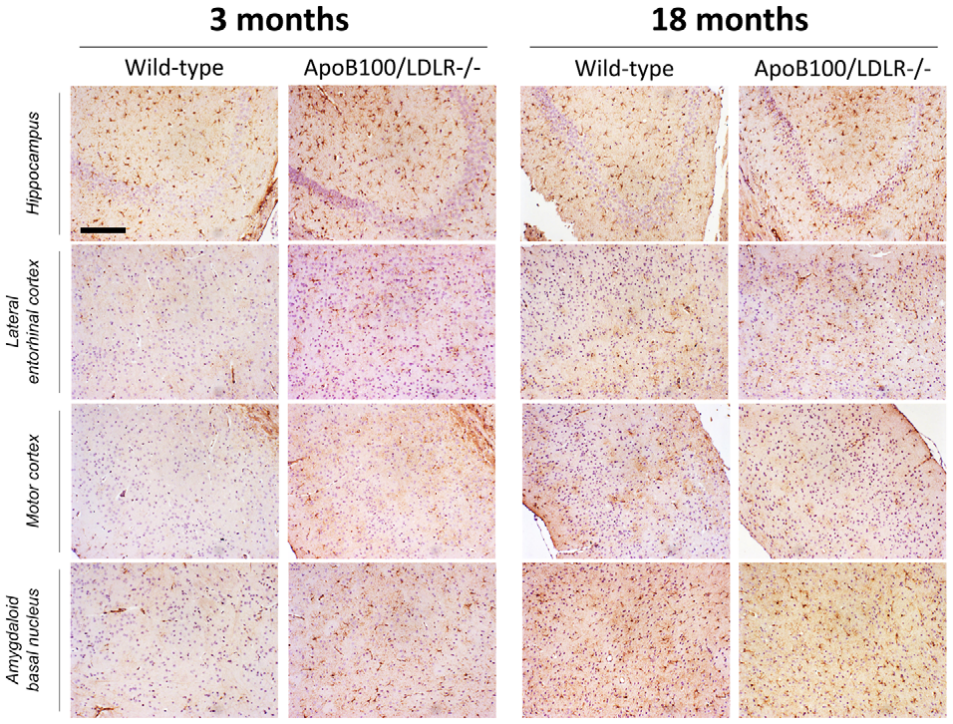
Astrogliosis in young and aged wild-type and transgenic mice. GFAP staining of coronal sections (1.58 mm from bregma) of the left (contralateral) hemisphere of a mouse brain from each of the age/genotype different groups. The hippocampus and three areas of the cortex are represented, showing several nuclei of the temporal lobe cortex, such as the lateral entorhinal, ectorhinal, the perirhinal and temporal-association cortices, the primary and secondary somatosensory cortices, the lateral parietal association cortex and visual cortex, and the cortical amygdaloidal nucleus and amygdalohippocampal area. Scale bar: 100 µm.

Brain samples were examined for signs of astrogliosis using GFAP polyclonal antibodies (Fig. 6). The 18-month-old wild type mice showed more activated astrocytes than the 3-month-old wild type mice. Astrogliosis was detected in the neuropil of the hippocampus and in several regions of the cortex and amygdala. These findings relate an increase in astrogliosis to ageing. Both the young and aged transgenic mice showed an increase number of activated astrocytes in the hippocampus, in the entorhinal and motor-sensorial area of the cortex, and in the basal amygdaloidal nucleus.

#### Cerebral β-amyloidosis

**Figure 7:**
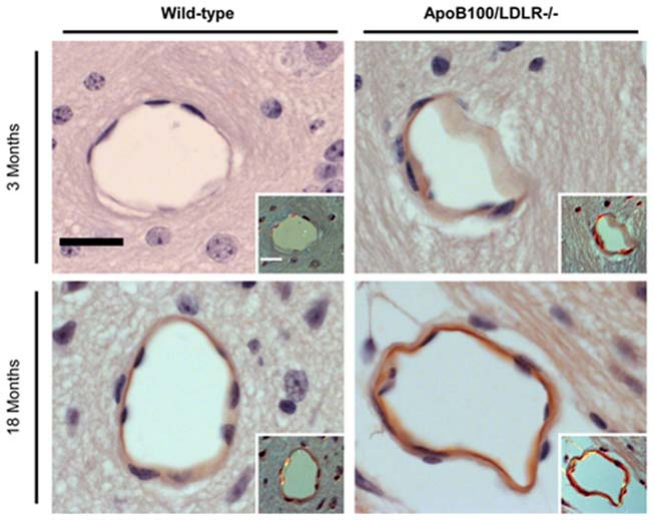
β-amyloidosis in the cerebral vessels of young and aged wild-type and transgenic mice. Congo red staining of coronal sections from each of the age/genotype different groups (1.62 mm from bregma). β-amyloid deposition can be seen in a vessel wall viewed under regular and polarized light (**insets**); note the apple-green birefringence specific to amyloid. Scale bars: 11 µm.

Aβ deposits appeared mostly as apple-green birefringent amyloid deposits restricted to the vessel walls (Fig. 7). Congo red staining revealed age-dependent cerebral β-amyloidosis in wild-type mice: no Aβ immunoreactive deposits were detected in the young mice, but were detected in the aged mice, mainly in the hypothalamus and occasionally in cortical areas (Fig. 7). However, in the young transgenic mice, Aβ deposits were detected in the vessel walls similar to those found in the aged wild-type mice. After 18 months, the Aβ deposits in the hypothalamus and cortical areas increased significantly in number and birefringence intensity in the transgenic mice (Fig. 7).

**Figure 8:**
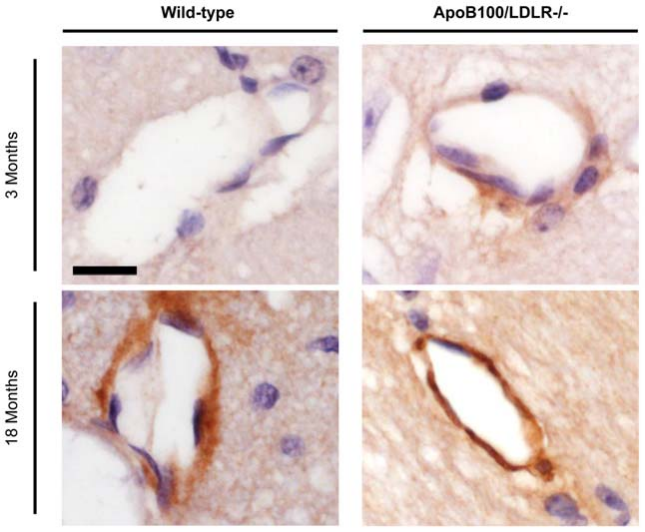
Aβ immunodetection in the cerebral vessels of young and aged wild-type and transgenic mice. Immunohistochemistry against Aβ of coronal sections from each of the age/genotype different groups. Scale bar: 11 µm.

To definitely confirm the Aβ deposits in the vessel walls, an immunohistochemical analysis using the monoclonal 4G8 antibody was performed (Fig. 8). Again, cerebral β-amyloidosis was dependent on genotype and age. Aβ immunoreactive deposits were not detected in the young wild-type mice. On the contrary, young transgenic mice presented specific Aβ deposits in the vessel walls, and the signal intensities were clearly increased in aged wild-type and transgenic mice.

## Discussion

Clinical and epidemiological evidence suggest that individuals with elevated midlife plasma cholesterol are at increased risk of dementia and AD [1], [2], [3], [4]. However, while much attention has been focused on the effect of cholesterol in murine AD models, little attention has been paid to neurodegeneration in hypercholesterolaemic mice; most studies have focused on the accumulation of amyloid plaques [7], [8], [9]. It remains unclear whether plasma cholesterol levels increase the risk of AD via the transport of cholesterol into the brain, or via indirect mechanisms such as an increase in cholesterol metabolites which then enter the brain. Either may be able to trigger Aβ production [23], and therefore cerebrovascular disease [24]. To investigate this, the present study examined the changes during aging in plasma cholesterol concentration along with the neuropathological and cognitive and psychomotor alterations in a hypercholesterolaemic mouse model (apoB100/LDLR-/-) that mimics human FH [13].

The main finding of this work was the direct association between FH and behavioural impairment in young hypercholesterolaemic mice. These results are in line with those of epidemiological studies showing that the proportion of patients with FH showing abnormal cognitive function and meeting the criteria for MCI is significantly higher than in controls [4]. Patients with FH are exposed to higher cholesterol levels from birth, and it is reported that hypercholesterolaemia may be an early risk factor for AD [2], [4]. Moreover, in patients with FH, it is very common for the LDLRs that participate in synapse maintenance to be dysfunctional, a pathogenic sign of AD [6]. Over 600 mutations causing FH in humans have been identified [25], and several polymorphisms in the LDLR gene have been associated with AD [26]; the potential progress from MCI to AD in people with these genetic backgrounds should be monitored.

The transgenic mice used in the present study are characterized by their deficiency in LDLRs and in their inability to splice apoB mRNA. This results in markedly elevated LDL cholesterol and apoB100 levels (thus providing a model of human FH) that develop extensive atherosclerosis when fed a chow diet [13]. This model is superior to previous hypercholesterolaemic models in which mice were only deficient in LDLRs, that had only mildly elevated LDL cholesterol levels and little atherosclerosis [27], or that were based exclusively on the overexpression of apoB100 and no alteration in plasma total cholesterol [28]. It should be noted that, in the present work, the transgenic mice were subjected to no pharmacological treatment or hypercholesterolaemic diet. With respect to most of the variables analysed, the young mice showed profiles similar to those of aged normal old mice, indicating that the hypercholesterolaemia of the former simulates accelerated aging and induces cognitive and psychomotor impairment. Independent of age the transgenic mice had high plasma TG, TC, FC and EC concentrations. Moreover, the plasma LDL-cholesterol fraction was dramatically increased compared to the control mice, as was apoB production, while the plasma HDL-cholesterol concentration was much lower.

An inverse relationship has been reported between plasma HDL-cholesterol and cerebral β-amyloid load in Tg2576 mice fed an atherogenic diet [10], and it has been shown that HDL-cholesterol increases the degradation of Aβ by microglia *in vitro* [29]. Additionally, the elevation of plasma TG precedes amyloid deposition in the murine Tg-CRND8, APP/PS1 and Tg-SwDI/B AD models [30]. In AD patients, LDL-cholesterol and apoB concentrations are increased, and correlate with brain Aβ concentrations [31], [32], [33]. In general, elevated TC, LDL and TG characterise the lipid profiles of AD subjects, while high HDL-cholesterol levels in elderly individuals are associated with a reduced risk of the disease [34], [35], [36], [37]. These results in AD-mouse models and AD patients are highly coincident with the findings in the present transgenic mice, and together indicate that changes in the plasma cholesterol profile might be involved in the initiation and progress of the neurodegenerative process. Moreover, since the increase in TC, FC, EC, LDL, apoB and TG, plus the reduction in HDL-cholesterol, are predictors of cognitive impairment, they might be proposed as translational biomarkers for MCI and/or AD.

The behavioural deficits observed in the aged wild-type and young and old transgenic mice correlated well with their high plasma cholesterol concentrations. Young transgenic mice showed a loss of muscle strength compared to the young wild-type mice. Moreover both the young and old transgenic mice showed longer periods of immobility and fewer rearings than wild-type mice in the open field test. This indicates that global motor activity is compromised as a consequence of hypercholesterolaemia. These results are in line with the observed decline in strength in older people – which is also associated with an increased risk of AD [38].

At 18 months of age, both genotypes experienced significant temporal and spatial memory deficits. However, the young transgenic mice also showed a significant decline in temporal memory (compared to young wild-type mice), indicating that these animals suffer an MCI-like condition. These results are in line with those recorded in humans with vascular risk factors for AD [39], [40] and with the cognitive impairment that characterizes the clinical progression of AD.

The present results relating to hypercholesterolaemia and cognitive and psychomotor impairments are in line with those of previous reports indicating cholesterol-induced memory deficits in transgenic mice expressing human mutant APP [41], with those of studies on hypercholesterolaemia-induced memory dysfunction in LDLR-/- mice [11], [33], and with those on cognitive impairment in rats fed high saturated fat diets [42]. However, the effect of the aging, which is critical in the context of neurodegenerative diseases, was not taken into account in these earlier studies.

Neuropathological analyses were undertaken to study the potential correlation between cognitive and psychomotor impairment and the status of different brain areas. Different signs of chronic neuronal damage were recorded in the 3-month-old transgenic mice, including neuritic dystrophy, oxidative damage and astrogliosis. The presence of neuronal damage in the entorhinal cortex and the basal amygdaloid nucleus in these young mice explains the impairment detected in their temporal memory. In addition, clear signs of oxidative damage were detected in the neurons of the sensory-motor cortex of the young transgenic mice, which explains the accelerated motor and coordination impairment in them. In the hippocampus, however, astrogliosis and oxidative damage was more dependent on age than genotype.

To relate the potential involvement of atherosclerosis and cerebrovascular damage to the behavioural and neuropathological changes observed in the transgenic mice, the aorta was examined for atherosclerotic plaques and different brain areas for β-amyloidosis. Atherosclerosis has been associated with AD and cerebral angiopathy in humans [43], [44], and recent findings indicate that vascular risk factors and neurovascular dysfunction play integral roles in the pathogenesis of AD [45]. Moreover, a relationship is reported between aortic atherosclerosis and brain Aβ deposits and learning deficits in transgenic Tg2576 mice fed an atherogenic diet [41]. Cholesterol may affect β-amyloidosis by modulating the proteolytic processing of APP and/or subsequent amyloid formation and deposition [9], [46]. In humans, Aβ peptide accumulates in the core of senile plaques and in blood vessel walls; in fact, >90% of human amyloid plaques are in direct contact with capillaries [47]. Moreover, it has been shown that cerebral amyloid angiopathy (CAA) caused by Aβ deposition has a pathogenic role in dementia (reviewed in [48]). Indeed, many of the mouse models for genetic AD that harbour different human APP mutations have also been shown to exhibit CAA [49], [50], although the overexpression of APP alone causes no Aβ deposition in most mouse lines [51], and mouse app does not generate amyloid plaques. Interestingly, the present transgenic mice showed early cerebrovascular β-amyloidosis that was exacerbated with age, in the same way that, in human mutant APP mouse models, a close relationship is seen between high plasma cholesterol and brain vessel amyloidosis [49], [50]. In the present transgenic mice, the severity of the cognitive and psychomotor deficits were closely associated with an increase in amyloidosis, suggesting that exacerbation of this event, rather than atherosclerosis, may be a potential mechanism for its detrimental effect on motor activity and memory. The elevated cholesterol concentrations of the transgenic mice may induce damage in the brain vasculature via the deposition of Aβ in the blood vessels. The eventual obliteration of lumens [52] would lead to the dysfunction of the affected areas. Certainly, increased plasma cholesterol concentrations correlate with increased Aβ accumulation in human brains [53].

In summary, hypercholesterolaemia early in the life of the ApoB100/LDLR-/- mice is associated with neuritic dystrophy, oxidative damage, astrogliosis and vascular β-amyloidosis, leading to neuronal damage and eventually psychomotor and cognitive impairment. The present results suggest that changes in plasma lipid homeostasis precede cognitive and psychomotor decline, and potentiate the cerebral β-amyloidosis that occurs with aging. These transgenic mice could provide an excellent model for the study of sporadic AD in humans.
